# Esophageal cancer localization by sagittal computed tomography images and endoscopic measurement

**DOI:** 10.1002/kjm2.12834

**Published:** 2024-04-17

**Authors:** Jen Yang, Wei‐Lun Chang, Forn‐Chia Lin, Nan‐Tsing Chiu

**Affiliations:** ^1^ Department of Medical Imaging National Cheng Kung University Hospital, College of Medicine, National Cheng Kung University Tainan Taiwan; ^2^ Department of Internal Medicine National Cheng Kung University Hospital, College of Medicine, National Cheng Kung University Tainan Taiwan; ^3^ Department of Oncology National Cheng Kung University Hospital, College of Medicine, National Cheng Kung University Tainan Taiwan

Accurate localization of esophageal tumors is essential for effective radiotherapy planning, which aims to reduce geographic miss and improve loco‐regional disease control. Traditional imaging modalities like computed tomography (CT) and positron emission tomography (PET) sometimes fail to detect small esophageal tumors due to their limited spatial resolutions. In clinical practice, a common approach to overcome this limitation is to use the carina‐incisor distance (CID) assumption of 25 cm to locate tumors based on endoscopic measurements.^1^ However, this method can be inaccurate due to individual variations in CID.^2^ Previous research has shown a high level of agreement between fludeoxyglucose‐18 (FDG) PET/CT and endoscopic measurements in determining the distance from the incisors to the proximal margins of esophageal cancer.^3^

In this study, we aimed to improve tumor localization accuracy for esophageal cancer patients undergoing radiotherapy with CT invisible esophageal cancer. We compared a new method using sagittal CT images with endoscopic measurements to the traditional method based on the assumption of a carina‐incisor distance (CID) of 25 cm.

We retrospectively enrolled 36 esophageal cancer patients who had pretreatment endoscopy and FDG PET/CT with visible esophageal tumor. The mean distance from the incisors to the proximal and distal tumor margins measured during endoscopy was 27.7 ± 6.4 cm (range, 17.0–40.0 cm) and 33.4 ± 6.0 cm (range, 21.0–42.5 cm), respectively. The interval between FDG PET/CT and endoscopy was 11.5 ± 7.1 days (range, 0–29 days; Table 1).

**TABLE 1:** Summary of patient characteristics (*n* = 36).

No.	Age	Gender	Tumor location	T	N	M	Histologic subtype	Treatment
1	65	M	Upper thoracic	3	3	0	Squamous cell carcinoma	1
2	57	M	Lower thoracic	2	0	0	Squamous cell carcinoma	2
3	72	M	Upper to middle thoracic	3	3	0	Squamous cell carcinoma	1
4	61	M	Middle to lower thoracic	3	3	1	Squamous cell carcinoma	4
5	53	M	Lower thoracic	2	2	0	Squamous cell carcinoma	2
6	49	F	Lower thoracic	1	0	0	Squamous cell carcinoma	3
7	58	M	Cervical	3	3	1	Squamous cell carcinoma	1
8	47	M	Middle to lower thoracic	3	2	0	Squamous cell carcinoma	2
9	50	M	Middle to lower thoracic	3	3	1	Squamous cell carcinoma	1
10	59	M	Upper thoracic	3	2	0	Squamous cell carcinoma	2
11	63	M	Upper to lower thoracic	3	3	1	Squamous cell carcinoma	1
12	61	M	Upper thoracic	3	3	0	Squamous cell carcinoma	1
13	52	F	Middle thoracic	3	3	0	Squamous cell carcinoma	2
14	67	M	Cervical	3	2	0	Squamous cell carcinoma	3
15	81	M	Lower thoracic	3	2	0	Adenocarcinoma	1
16	52	M	Lower thoracic	3	2	0	Squamous cell carcinoma	2
17	55	M	Lower thoracic	3	0	0	Squamous cell carcinoma	1
18	58	M	Middle thoracic	3	2	1	Squamous cell carcinoma	1
19	63	M	Upper to middle thoracic	3	3	1	Squamous cell carcinoma	1
20	48	M	Lower thoracic	3	3	1	Squamous cell carcinoma	1
21	69	M	Lower thoracic	3	3	1	Squamous cell carcinoma	1
22	57	M	Middle thoracic	3	2	0	Squamous cell carcinoma	2
23	44	M	Lower thoracic	3	2	0	Squamous cell carcinoma	1
24	52	M	Lower thoracic	1	0	0	Adenocarcinoma	2
25	65	M	Lower thoracic	3	3	1	Squamous cell carcinoma	1
26	54	M	Upper thoracic	3	2	0	Squamous cell carcinoma	2
27	61	M	Middle thoracic	1	0	0	Squamous cell carcinoma	1
28	42	M	Lower thoracic	3	1	0	Adenocarcinoma	3
29	63	M	Upper to middle thoracic	4	3	0	Squamous cell carcinoma	1
30	69	M	Middle to lower thoracic	2	2	0	Squamous cell carcinoma	4
31	51	M	Middle to lower thoracic	3	3	0	Squamous cell carcinoma	2
32	56	M	Upper to middle thoracic	3	3	0	Squamous cell carcinoma	1
33	50	M	Upper to middle thoracic	3	3	0	Squamous cell carcinoma	1
34	58	M	Lower thoracic	2	0	0	Squamous cell carcinoma	3
35	47	M	Upper to middle thoracic	3	3	0	Squamous cell carcinoma	1
36	83	F	Middle to lower thoracic	3	0	0	Squamous cell carcinoma	1

*Note*: Treatment: 1 = Concurrent chemoradiotherapy, 2 = Concurrent chemoradiotherapy with subsequent operation, 3 = Operation alone, 4 = Chemotherapy alone.

For our new method, we drew a polyline on sagittal CT images from PET/CT to mimic the endoscope's pathway from the incisor to the tumor margins. For the traditional method, we assumed a CID of 25 cm to locate the tumor margins on the transverse CT images from PET/CT. We then compared the accuracy of these methods using FDG PET/CT as the reference.

The results showed that the mean absolute distances between the reference and the new method were significantly smaller than those between the reference and the traditional method. Specifically, the mean absolute distances for the proximal and distal margins were 1.46 ± 1.00 cm (range, 0.09–3.97 cm) and 1.23 ± 1.13 cm (range, 0.02–5.44 cm), respectively, for the new method, compared with 3.55 ± 1.87 cm (range, 0.11–6.84 cm) and 3.68 ± 1.63 cm (range, 0.43–6.85 cm), respectively, for the traditional method. The differences of the two methods in absolute distances for both proximal (mean difference = −2.09 cm, *p* < 0.001) and distal (mean difference = −2.44 cm, *p* < 0.001) margins were significant. Bland–Altman plot analysis was also conducted to assess the agreement between the reference (FDG PET/CT) and the two CT methods (Figure 1).

**FIGURE 1:**
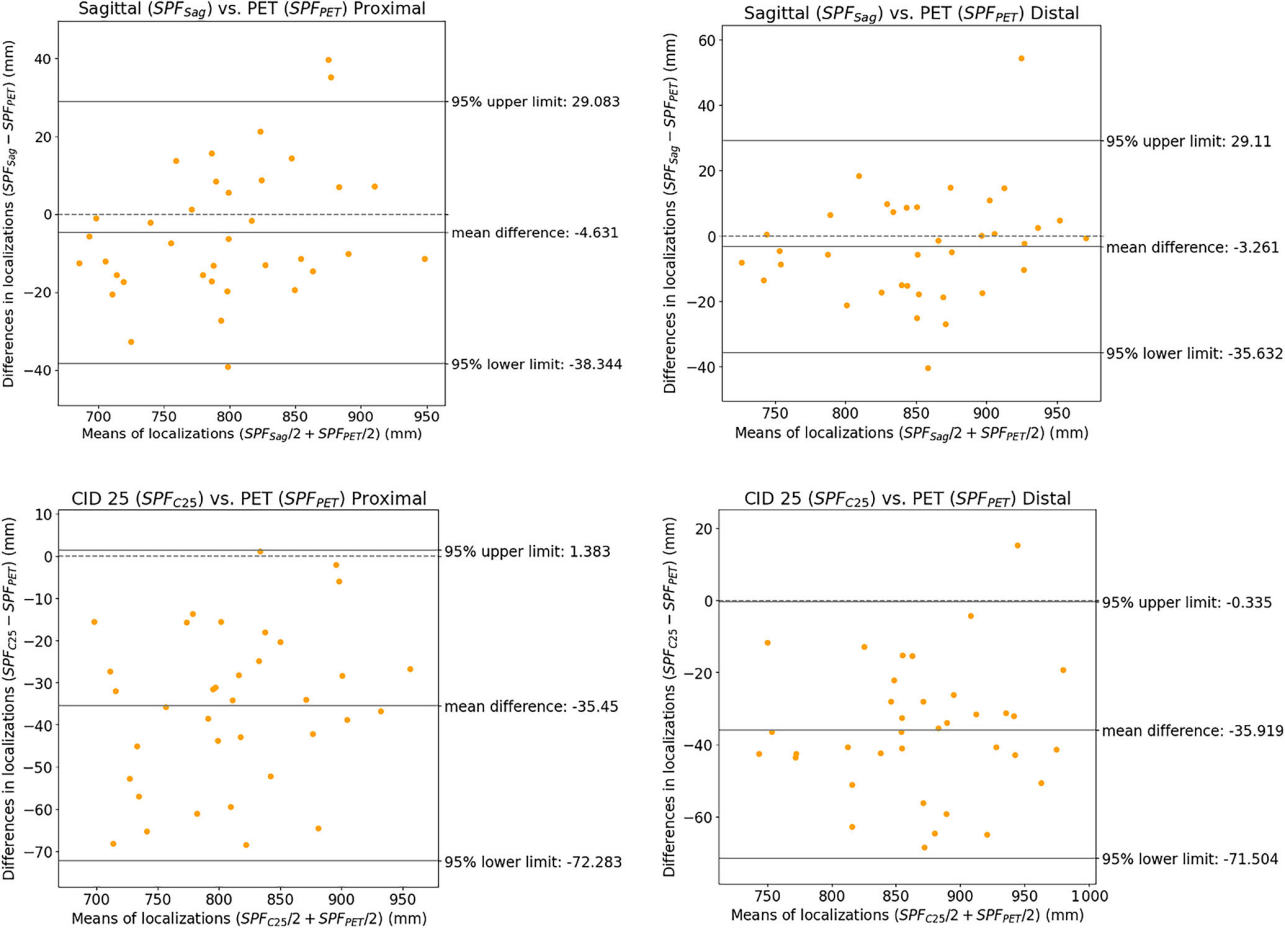
Bland–Altman (B‐A) plot for proximal and distal margins determined in different ways versus positron emission tomography (PET)/computed tomography (CT)‐labeled tumor locations. B‐A plot, Bland–Altman plot; Sagittal (d_Sag_), localization of tumor margins by sagittal CT images; carina‐incisor distance (CID) 25 (d_C25_), localization of tumor margins by assumed CID of 25 cm; PET (d_PET_), localization of tumor margins by PET/CT.

Additionally, the Jaccard index (JI), which measures the similarity between the methods and the PET/CT reference, was significantly higher for the new method (0.56 ± 0.28) than for the traditional method (0.25 ± 0.23), indicating better consistency with the reference. The JI was zero in 2 (5.56%) patients for the new method and in 11 (30.56%) patients for the traditional method.

We also assessed the coverage of the FDG PET/CT tumor with the addition of a longitudinal margin to the tumor delineated by the two methods. The results showed that the FDG PET/CT tumor was fully covered with an added margin of 1, 2, 3, and 4 cm in 36.1% (95% CI: 22.5%–52.4%), 86.1% (95% CI: 71.3%–93.9%), 91.7% (95% CI: 78.2%–97.1%), and 97.2% (95% CI: 85.8%–99.5%) of cases, respectively, for the new method. In contrast, the traditional method showed lower coverage rates.

Our study highlights the superiority of using sagittal CT images with endoscopic measurements over the traditional assumption of a 25 cm carina‐incisor distance for localizing esophageal tumors in radiotherapy planning. This method offers a more accurate approach, especially for small or early‐stage tumors that are often invisible on conventional CT or PET scans.^4^, ^5^ By closely mimicking the endoscopic pathway, we achieved better coverage of the macroscopic disease, reducing the risk of geographic miss in radiation treatment.

However, this method has its limitations. First, it relies on the subjective interpretation of the endoscope's pathway and may be influenced by anatomical variations and differences in patient positioning during imaging. Second, our study, with a sample size of 36 patients, may not fully represent diverse clinical scenarios, potentially limiting the broader applicability of the findings. Third, the study did not examine how this method compares with advanced imaging technologies like magnetic resonance imaging (MRI), with or without diffusion‐weighted imaging, or enhanced PET/CT protocols that might yield more precise tumor localizations. Fourth, the study recognizes but does not fully explore the practical challenges in adopting the new method clinically. These include the need for specialized training for radiologists, the time required to meticulously draw polylines on sagittal CT images, and the method's integration into existing radiotherapy workflows. Moreover, usability aspects, such as the user interface of the analysis software, could influence both the efficacy and the acceptance of this method in a clinical setting. Despite these challenges, our findings suggest that this approach could significantly improve the precision of radiotherapy targeting for esophageal cancer patients.

In conclusion, our study provides a promising alternative for tumor delineation in esophageal cancer, especially for cases where traditional imaging falls short. Further research and prospective studies are needed to refine this technique and confirm its benefits in clinical practice.

## CONFLICT OF INTEREST STATEMENT

All authors declare no conflict of interest.

## References

[1] Czito BG , Palta M , Willett CG . Esophageal cancer. Perez and Brady's principles and practice of radiation oncology. 7th ed. Philadelphia: Wolters Kluwer; 2019. p. 1206–1243.

[2] Rice PF , Crosby TL , Roberts SA . Variability of the carina‐incisor distance as assessed by endoscopic ultrasound. Clin Oncol (R Coll Radiol). 2003;15:383–385. 14570085 10.1016/s0936-6555(03)00115-8

[3] Hsu SW , Chang JS , Chang WL , Lin FC , Chiu NT . Measuring distance from the incisors to the esophageal cancer by FDG PET/CT: endoscopy as the reference. BMC Gastroenterol. 2022;22:126. 35300618 10.1186/s12876-022-02206-z PMC8928607

[4] Desai RK , Tagliabue JR , Wegryn SA , Einstein DM . CT evaluation of wall thickening in the alimentary tract. Radiographics. 1991;11:771–783. discussion 784. 1947313 10.1148/radiographics.11.5.1947313

[5] Hong SJ , Kim TJ , Nam KB , Lee IS , Yang HC , Cho S , et al. New TNM staging system for esophageal cancer: what chest radiologists need to know. Radiographics. 2014;34:1722–1740. 25310426 10.1148/rg.346130079

